# Whole-Genome Sequencing of a *Pandoravirus* Isolated from Keratitis-Inducing *Acanthamoeba*

**DOI:** 10.1128/genomeA.00136-15

**Published:** 2015-03-26

**Authors:** M. H. Antwerpen, E. Georgi, L. Zoeller, R. Woelfel, K. Stoecker, P. Scheid

**Affiliations:** a Bundeswehr Institute of Microbiology, Munich, Germany; b Laboratory of Medical Parasitology, Central Institute of the Bundeswehr Medical Service, Koblenz, Germany

## Abstract

Following the recent discovery of two *Pandoravirus* species in 2013, a previously described endocytobiont isolated from the inflamed eye of a patient with keratitis was subjected to whole-genome sequencing (WGS). Here, we present the complete genome sequence of a new *Pandoravirus* isolate.

## GENOME ANNOUNCEMENT

In 2008, Scheid et al. (1) described an unknown endocytobiont isolated from the Acanthamoeba strain LaHel culture recovered from the inflamed eye of a patient with keratitis. Triggered by the discovery of two *Pandoravirus* species (2), the endocytobiont was reinvestigated based upon similar morphology.

For whole-genome sequencing, short reads were produced by IonTorrent PGM technology (Life Technologies, Darmstadt, Germany) and long reads were produced by PacBio RS II technology (Pacific Biosciences, Menlo Park, CA, USA).

For this short-read library 1 µg of DNA was processed with the Ion fragment library kit for 400 bp-chemistry (IonXpress template kit). Raw data (q15w10) were mapped against Acanthamoeba castelanii strain Neff, Enterobacter cloacae WSU1, and Flavobacterium johnsonii UW101 to eliminate contaminating sequences. The remaining reads were assembled using GS Assembler of Newbler 2.6 software.

In parallel, 3 µg of DNA were used to generate an SMRTBell library. The library was sequenced using two flow cells with P4-C2 chemistry (Pacific Biosciences). Raw-data reads were used to generate long scaffolds in combination with short-read contigs from IonTorrent data using SMRTAnalysis software 2.1. Gaps were closed by two consecutive runs of PBJelly (3). The genome was closed after applying progressiveMauve (4). This consensus sequence was used to map original reads from both approaches to verify the calling and for correcting potential miscalls.

Functional annotation was performed using the GeneMarkS software (5, 6) with default settings. Predicted open reading frames (ORFs) were translated and each protein sequence was compared with the NCBI protein database using BLASTp and CLC Genomics Workbench 7.0.4 (CLC bio). Best hits were assigned to their corresponding ORFs by in-house scripts. ORFs showing no significant hit were labeled as hypothetical proteins. tRNA prediction was performed using the tRNAscan-SE Search Server (7). The G+C content was calculated using an in-house Python script.

From IonTorrent PGM sequencing, 2,113,783 reads were generated and *de novo* aligned to 4,359 contigs > 1,000 bp; 98.74% of all nucleotides were assigned to a quality of Q40.

Of the 154,070 reads, only 11,675 PacBio reads with an average length of 5,755 bp were mapped to *de novo* assembled contigs of short-read sequencing. The average coverage depth of concatenated reads was 62-fold. The continuous nucleotide sequence was 2,243,109 bp with a G+C-content of 60.66%. A BLASTn search against the nonredundant database showed a nucleotide sequence identity of 89% to Pandoravirus dulcis and 85% to Pandoravirus salinus.

Annotation using GeneMarkS (5, 6) discovered 1,902 putative coding sequences comprising 1,339 hypothetical proteins (70%) and 220 MORN- and ankyrin-repeat structures. Only 1,389 of the discovered proteins have homologues within other members of genus *Pandoravirus*. tRNAScan discovered tRNA-Proline as the only tRNA present in this genome. No genes coding for amino-acid-tRNA ligases were found.

Comparing the genomes on the protein level of B-family DNA polymerases and DNA-directed RNA polymerase II with homologous sequences of the viral orthologous genes (NCVOG) of the nucleocytoplasmic large DNA viruses revealed clustering of the isolate’s sequence to the recently published sequences of P. dulcis and P. salinus.

As 93% of the *Pandoravirus* genes could not be assigned to known functions, many new details on this unique group of organisms are expected in future studies.

### Nucleotide sequence accession number.

The genome sequence of this new *Pandoravirus* isolate was submitted to NCBI and is accessible in its first version with the accession no. KP136319.
